# Use of the DEKA Arm for amputees with brachial plexus injury: A case series

**DOI:** 10.1371/journal.pone.0178642

**Published:** 2017-06-19

**Authors:** Linda Resnik, Christopher Fantini, Gail Latlief, Samuel Phillips, Nicole Sasson, Eve Sepulveda

**Affiliations:** 1 Providence VA Medical Center, Providence, RI, United States of America; 2 Health Services, Policy and Practice, Brown University, Providence, RI, United States of America; 3 Amputation System of Care—Northeastern Region, United States Department of Veterans Affairs, James J. Peters VA Medical Center, Bronx, NY, United States of America; 4 Southeast Regional Amputation Center, Tampa, FL, United States of America; 5 University of South Florida, College of Medicine, Department of Neurology, Division of PM&R, Tampa FL, United States of America; 6 HSR&D/RR&D Center of Innovation on Disability and Rehabilitation Research (CINDRR), Tampa, FL, United States of America; 7 VA New York Harbor Healthcare System, New York, New York, United States of America; 8 Rehabilitation Medicine, New York University School of Medicine /Rusk Institute, New York, New York, United States of America; Shanghai Jiao Tong University, CHINA

## Abstract

**Objective:**

Patients with upper limb amputation and brachial plexus injuries have high rates of prosthesis rejection. Study purpose is to describe experiences of subjects with transhumeral amputation and brachial plexus injury, who were fit with, and trained to use, a DEKA Arm.

**Methods:**

This was a mixed-methods study utilizing qualitative (e.g. interview, survey) and quantitative data (e.g. self-report and performance measures). Subject 1, a current prosthesis user, had a shoulder arthrodesis. Subject 2, not a prosthesis user, had a subluxed shoulder. Both were trained in laboratory and participated in a trial of home use. Descriptive analyses of processes and outcomes were conducted.

**Results:**

Subject 1 was fitted with the transhumeral configuration (HC) DEKA Arm using a compression release stabilized socket. He had 12 hours of prosthetic training and participated in all home study activities. Subject 1 had improved dexterity and prosthetic satisfaction with the DEKA Arm and reported better quality of life (QOL) at the end of participation. Subject 2 was fit with the shoulder configuration (SC) DEKA Arm using a modified X-frame socket. He had 30 hours of training and participated in 3 weeks of home activities. He reported less functional disability at the end of training as compared to baseline, but encountered personal problems and exacerbation of PTSD symptoms and withdrew from home use portion at 3 weeks. Both subjects reported functional benefits from use, and expressed a desire to receive a DEKA Arm in the future.

**Discussion:**

This paper reported on two different strategies for prosthetic fitting and their outcomes. The advantages and limitations of each approach were discussed.

**Conclusion:**

Use of both the HC and SC DEKA Arm for patients with TH amputation and brachial plexus injury was reported. Lessons learned may be instructive to clinicians considering prosthetic choices for future cases.

## Introduction

Traction injury of the brachial plexus with complete lesion can cause total and permanent paralysis of the upper extremity leading to shoulder subluxation and complete loss of function in the affected limb. Although optimal treatment is still debated, [1] some patients undergo delayed amputation to remove the flail, insensate extremity. [2] Given that pain in the shoulder and the weight of the prosthesis is an impediment to successful prosthesis use, some recommend that amputation be accompanied by arthrodesis of the shoulder.[3, 4] Thus, many, but not all, patients with this type of injury ultimately have surgical fusion of the shoulder joint to correct instability and place the extremity in a more functional position. [1]

Several reports suggest that patients with amputation secondary to brachial plexus injuries are more likely to reject or abandon prosthesis use. In 1961 Yeoman reported on a series of 15 patients with both amputation and arthrodesis that only 40% (6/15) were regular body powered prosthesis users, 20% (3/15) were occasional users and 40% (6/15) non-users.[5] Another case series, consisting of 13 patients, reported that prosthetic usage varied by limb dominance prior to the amputation. [2] Of those with amputation to the dominant extremity, only 2/13 (29%) used a prosthesis with active controls, while 3 (43%) used a cosmetic prosthesis, and 1/13 (14%) used a prosthesis without a terminal device for balance only. For those who lost a non-dominant extremity, 3/6 (50%) used an active prosthesis, 2/6 (33%) a cosmetic prosthesis, and 1/6(17%) a prosthesis without terminal device. Maldonado reported on 9 patients with brachial plexus injuries who were treated by elective amputation and reported that none utilized dynamically controlled prostheses, although 3 did wear passive aesthetic ones. [1] Rorabeck suggested that the likelihood of becoming a prosthesis user was increased if the amputation was performed within the first year of injury.[6]

The rates of prosthesis use amongst persons with brachial plexus injury are comparable to rates of use reported in the broader population of upper limb amputees. [7, 8] Rejection or abandonment of prostheses is a common problem for persons with transhumeral amputation, regardless of etiology. Many individuals with upper limb amputation abandon or reject their prostheses because they are not satisfied with available prosthetic options. Studies show that the rejection rate for prostheses varies with amputation level. Those with amputations at the transradial (TR) level have the lowest rate of rejection (6%), followed by transhumeral (TH) level (57%), and persons with shoulder disarticulation (SD) (60%).[8, 9]

Currently available prostheses fall short of restoration of full upper limb function.[10] At best, commercially available myoelectric prostheses offer 3 powered motions: elbow flexion and extension, wrist rotation and open/close of the terminal device. Some newer hands can be programmed to close in a range of different patterns; however, the same control input is used to open/close the hand, regardless of the selected grip pattern used. Patients with intact musculature and range of movement at the shoulder can utilize the shoulder joint to bring their terminal device into a variety of positions and orientations. However, patients with paralysis of the shoulder are unable to use their intact musculature to change the device position in space. Thus, shoulder paralysis and/or arthrodesis dramatically shrinks the functional envelope (or workspace) available to these upper limb prosthesis users. Loss of the shoulder’s 3 degrees of freedom (flexion/extension, abduction/adduction and humeral internal/external rotation) limits the functional activities that can be performed largely to the sagittal plane (where movements of the elbow can be made available), and requires the user to utilize extensive body compensation at the trunk to engage in many every day activities. Additionally, users may need to use their intact contralateral limb to preposition the prosthesis into humeral internal rotation when using the device close to their body. It seems likely that restoration of any of the lost degrees of freedom of the shoulder joint, in these cases, would increase functionality of an upper limb prosthesis.

One promising device that may be suitable for this population of patients is the DEKA Arm, a pre-commercial prosthesis approved for marketing by the FDA and expected to become commercially available in the near future. The DEKA Arm is available in several configurations to meet the needs of persons with transradial amputation (RC: radial configuration), transhumeral (HC: humeral configuration) and shoulder disarticulation or scapulothoracic amputation (SC: shoulder configuration). It offers more powered degrees of freedom and unique control options than any other device currently on the market. Thus, this device could be particularly beneficial for persons with amputation secondary to brachial plexus injury. The HC DEKA Arm, which may be appropriate for a transhumeral amputee with a shoulder arthrodesis, offers the following: powered elbow flexion and extension, powered humeral internal and external rotation (with an axis of rotation just proximal to the elbow joint), and powered wrist motion with compound movement, resulting from a canted axis, of wrist flexion with ulnar deviation and wrist extension with radial deviation. The HC DEKA Arm weighs 6.8 lbs.

The SC DEKA Arm, may be appropriate for those patients with transhumeral amputation as mentioned above, who might be unable to tolerate the weight of a prosthesis on a transhumeral socket. The SC Arm offers all of the features of the HC Arm and an additional two degrees of powered movement at the shoulder joint; flexion/extension and abduction/adduction; thereby enabling the users to reach away from their body as well as overhead. As mentioned above humeral rotation (a physiologic movement of the shoulder joint) is available with an axis of rotation above the elbow joint in the HC and SC DEKA Arm. The harnessing of an SC configuration transfers the weight of the 9.8 lb SC DEKA Arm to the torso.

The purpose of this case series is to describe the experiences of two subjects with transhumeral amputation and brachial plexus injury, who were fit with, and trained to use, a DEKA Arm as part of a larger VA Study. One subject had shoulder arthrodesis and was fitted with the transhumeral configuration of the DEKA Arm, and one did not have arthrodesis and was fit with the shoulder configuration.

## Methods

The parent study had two parts. Part A included in-laboratory training and supervised community based outings using the DEKA Arm; Part B involved a trial of home use with biweekly study visits/calls and monthly in-person visits to the study site. The study was approved by the Institutional Review Boards at the Providence VA Medical Center, the VA New York Health Harbor System and the James A. Haley VA Medical Center. All subjects participated in two baseline testing sessions (see tests and measure below) before beginning Part A activities. After baseline testing was complete, participants returned for prosthetic socket fitting and for set up of DEKA Arm controls (described for each subject below). Participants were then oriented to the DEKA Arm features and controls by the study Occupational therapist and used the DEKA Arm’s virtual reality training environment (VRE) with a standardized VRE protocol developed for the study.[11] Participants practiced controlling the prosthesis within the VRE for up to 4 hours, prior to wearing the activated DEKA Arm.[12] After VRE, training began with the activated DEKA Arm. Training activities progressed from simple unilateral tasks and drills, to complex bimanual activities and functional tasks. Most training took place in the occupational therapy clinic, however, participants also completed several OT-supervised community outings. The OT and study PI used their clinical judgment to decide when the participants’ progress had plateaued and training was complete. Part B involved up to 12 weeks of use of the DEKA Arm at home, with in-person re-assessments every 4 weeks.

### Subject 1

#### Case description

**Fig 1:**
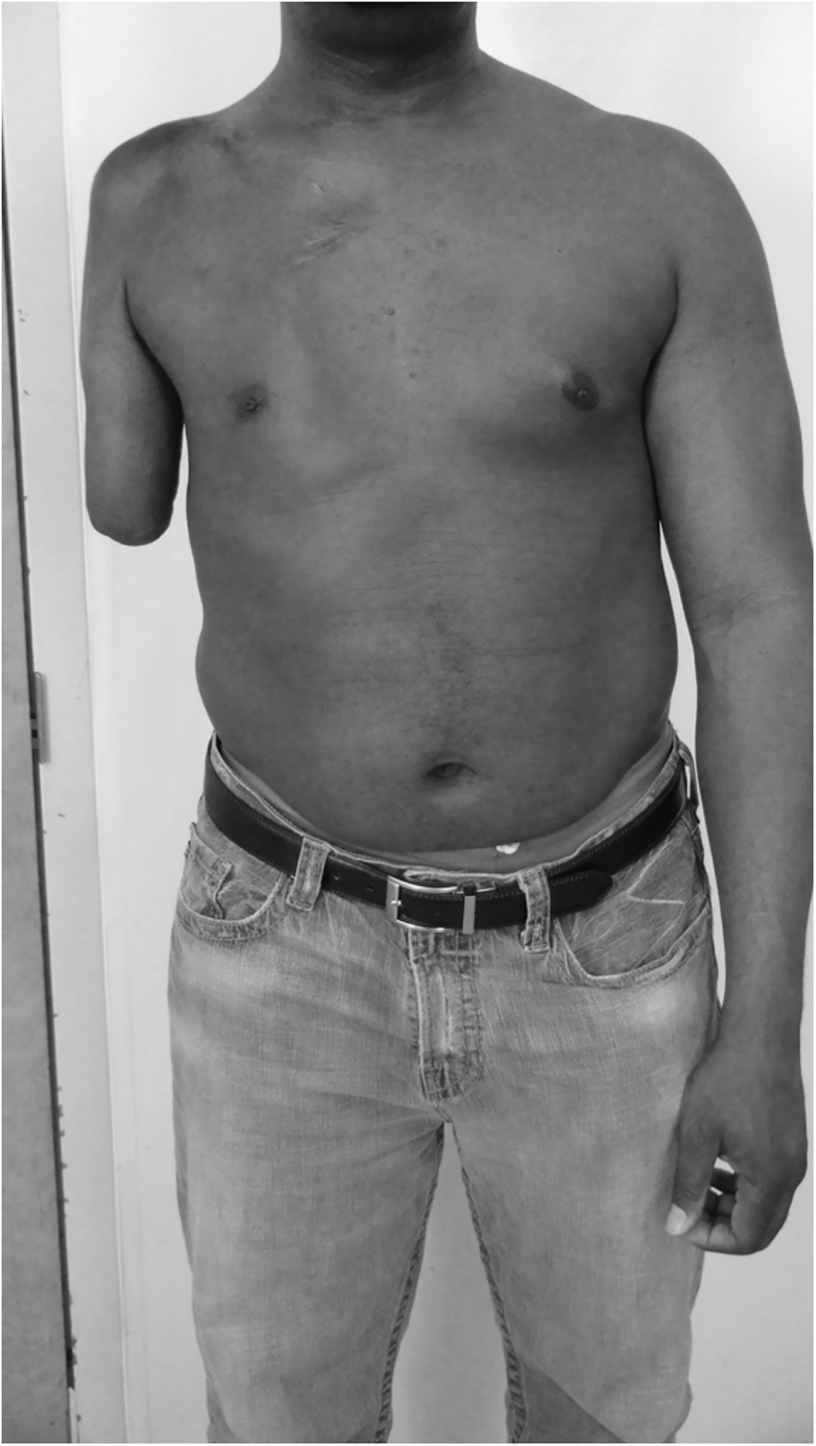
Subject 1 residual limb.

Subject 1 was a 46 year old black, non-Hispanic male Veteran with a unilateral right elective transhumeral amputation occurring more than 20 years prior (1994), that resulted from a brachial plexus injury and limb trauma sustained in a motorcycle accident in 1992. Previous to his amputation he was right hand dominant. The subject worked full-time and lived alone. The subject’s right shoulder was fused at the time of his accident. His shoulder musculature was atrophied due to brachial plexus injury. The residual limb (Fig 1) measured 27 cm in length from the acromion process to the end of the limb. He had mild phantom limb pain of 1/5 on Wong-Baker scale. [13]

#### Current prosthesis

**Fig 2:**
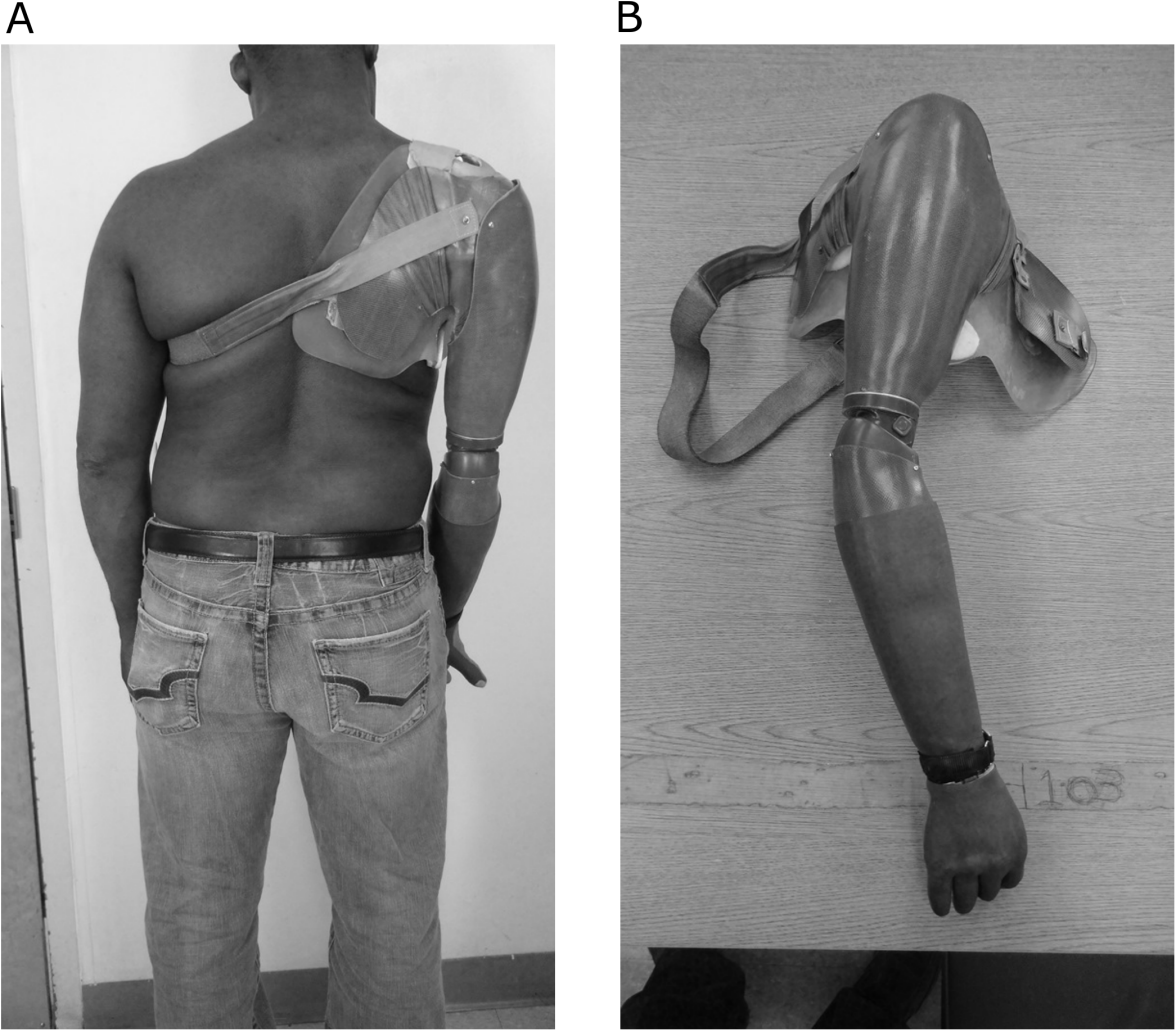
Subject 1 personal prosthesis.

The subject did not begin wearing a myoelectric prosthesis until 1998, 6 years after his injury and reported that he wore it full-time, approximately 12 hours per day. His most recent personal device, which he had owned for 2 years, consisted of a UTAH arm (elbow), with an electric wrist rotator, and a Motion Control hand. He controlled the device with 2 EMG sensors and used co-contraction to unlock his elbow, quick access control of the wrist, and slow access control of the hand (Fig 2). The socket was a conventional cylindrical socket with pectoral/scapula wings suspended by a shoulder saddle and chest strap. He described the device as “quite a bit” necessary both for maintaining his quality of life and maintaining his independence.

### Subject 2

**Fig 3:**
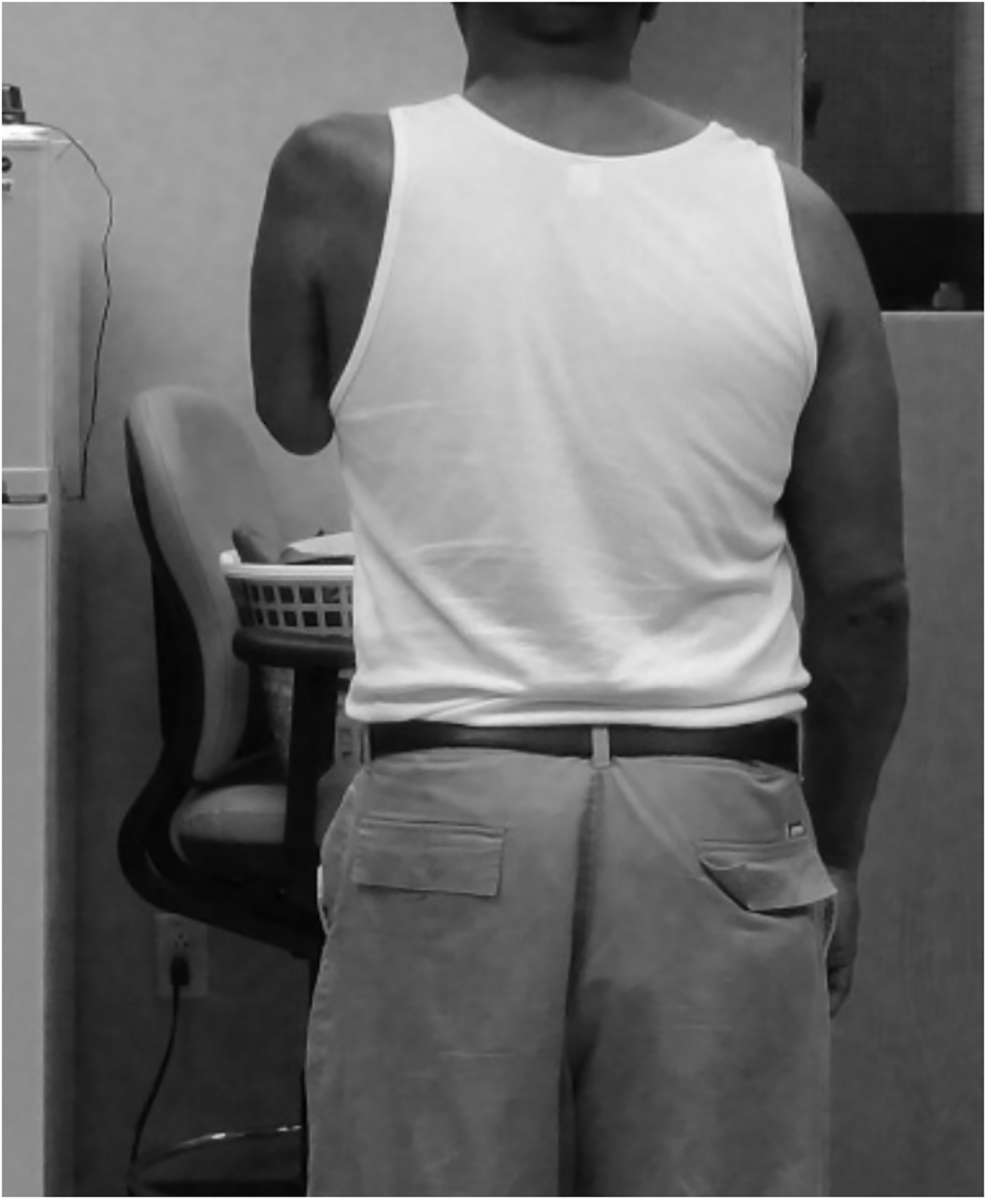
Subject 2 posterior view of residual limb.

Subject 2 was a 47 year old male Veteran of mixed race who was not employed at the time of study participation. He had a left unilateral transhumeral amputation due to trauma and brachial plexus injury in 2008. He had been right hand dominant before his amputation and maintained right hand dominance. He presented with a subluxed shoulder and flaccid residual limb. The residual limb measured 25 cm from the tip of the acromion to the distal end (Fig 3). He complained of phantom pain of 5/5 on the Wong Baker scale. The subject had a history of 2 traumatic brain injuries (TBIs) from prior motor vehicle accidents. Subject 2 did not use a prosthesis, although had tried a cosmetic prosthesis in the past. He also had tried an active body powered prosthesis, but found it unsuccessful and had abandoned it.

### Data collection and study measures

Both qualitative and quantitative data were collected throughout the study. Qualitative data included audio and video recorded during in-laboratory and testing sessions, and responses to open-ended survey questions and semi-guided interviews administered at the end of Parts A and B. Structured survey questions asked participants to indicate if they wanted to receive a DEKA Arm in the future; and to rate the following: their skill using the device; the weight of the device, and the comfort of the socket. Those who were prosthesis users prior to the study were also asked whether there were activities that they preferred doing with the DEKA.

Arm or vice versa. At the end of Part A participants were asked about the sufficiency of their training. At the end of Part B, participants who were prosthesis users were asked whether they preferred their own prosthesis to the DEKA Arm and to compare features of these devices (own prosthesis vs. DEKA Arm). They were also asked how necessary the DEKA Arm was for maintaining their quality of life, maintaining their independence, and improving quality of life and independence.

Quantitative data included structured survey responses and standardized measures, of activity limitation, prosthetic skill and spontaneity of use, disability, quality of life, prosthetic satisfaction and pain (see Table 1 for brief descriptions). These measures were collected at baseline, at the end of Part A and at monthly intervals during Part B. Self-report measures included: 1) the Upper-Extremity Functional Scale (UEFS),[14–17] 2) Patient-Specific Functional Scale (PSFS)[18]; 3) the Disabilities of the Arm, Shoulder and Hand Score (QuickDASH);[19, 20] 4) Trinity Amputation and Prosthesis Experience Scales (TAPES)[21]; 5) Quality of Life (QOL),[22] 6) the Community Reintegration of Service Members Computer Adaptive Test (CRIS-CAT),[23] and 7) the Wong-Baker FACES Pain Rating Scale (FACES).[24] Performance based measures included the Jebsen-Taylor Hand Function Test (JTHF),[25] 2) the Activities Measure for Upper-Limb Amputees (AM-ULA),[26] and 3) the University of New Brunswick Test of Prosthetic Function for Unilateral Amputees (UNB).[14, 27]

**Table 1:** List of measures and brief description.

Self-report measures	Construct	Item Content	Rating Criteria	Interpretation
Patient-Specific Functional Scale (PSFS)	Difficulty performing activities	5 self-selected activities difficult to do because of the amputation	Difficulty in performance	Higher scores indicates less difficulty
Upper-Extremity Functional Scale (UEFS)	Difficulty performing activities	Self-reported difficulty performing 23 everyday activities	Difficulty in performance	Lower scores indicates less difficulty
Upper-Extremity Functional Scale (Use)	Use of prosthesis	Self-reported use of the prosthesis during 23 everyday activities	Prosthesis use	Higher scores indicates higher proportion of activities done with prosthesis
Disabilities of the Arm, Shoulder and Hand Score (QuickDASH)	Disability	Self-reported functional difficulty (8 items) 3 items about sleep, sensation and pain	Performance difficulty and impairment severity	Higher scores indicate greater disability
The Community Reintegration of Service Members Computer Adaptive test (CRIS-CAT)		Computer adaptive testing measuring participation in life roles		Higher scores indicates better community integration
CRIS-CAT	Extent of participation		Frequency and amount	
CRIS-CAT	Perceived difficulty		Perceived limitations	
CRIS-CAT	Satisfaction		Satisfaction scale	
Quality of Life (QOL)	Quality of life	16 question items about quality of life	Satisfaction with quality of life	Lower scores indicate worse QOL
Trinity Amputation and Prosthesis Experience Scales (TAPES)	Prosthetic satisfaction	10 items satisfaction with prosthesis	Satisfaction	Higher scores indicate greater satisfaction
Won-Baker = Wong-Baker FACES Pain Rating Scale	Pain	Six faces showing levels of pain severity	Pain intensity	Higher scores indicates greater pain
**Performance Measures**				
Jebsen-Taylor Hand Function Test (JTHF)	Dexterity	7 separate tests of fine motor activities.	Performance speed; items / per second	Higher scores indicate better performance
University of New Brunswick Test of Prosthetic Function for Unilateral Amputees (UNB)		10 components of daily tasks that require bimanual engagement		
UNB	Prosthetic skill		Skillfulness of terminal device use.	Higher scores indicate better performance
UNB	Prosthetic spontaneity	10 components of daily tasks that require bimanual engagement	Spontaneity of engaging the prosthesis in activities	Higher scores indicate better performance
Activities Measure for Upper-Limb Amputees (AM-ULA)	Activity performance	18-everyday tasks	Task completion: speed, movement quality, skill and independence	Higher scores indicate better performance

### Data analyses

This was a mixed methods analyses which incorporated quantitative as well as qualitative findings. Self-report measures were graded using published algorithms.[28] Timed performance measures were graded by the study occupational therapist. The AM-ULA was graded by an off-site certified hand therapist who viewed videotaped testing sessions and was blinded to testing date. Results of quantitative metrics and surveys were summarized and described. Key subject impressions about their experiences, impressions of the prosthetic socket and the functionality of the DEKA Arm were gleaned from qualitative data sources including the open-ended survey questions and semi-guided interviews conducted at the end of each phase. Rich text examples were selected to illustrate subject perspectives.

## Results

### Subject 1

#### Prosthetic fitting and controls set-up

Though the amputation was at the TH level, due to the loss of active function of the residual limb, the research clinicians, in consultation with the subject, did consider the SC configuration of the DEKA arm for this case, to restore function at the shoulder level. However, due to the long length of the residual limb, they concluded that it would not have been possible to position the prosthetic shoulder joint close to the torso, beneath the end of the limb. This would have resulted in excessive asymmetry with the body and functional alignment issues with the prosthesis and created problems with fit of the subject’s clothing. In the team’s opinion, these costs outweighed the potential gain of shoulder movement. The team, including the subject, decided it was best to go with the TH DEKA Arm.

The fitting and controls set-up process for the initial socket took place over the course of 4 prosthetist visits. The subject was casted for and fit with a compression release stabilized CRS) check socket made of a rigid, clear, thermoplastic material (Vivak). [29] The CRS socket design is meant to minimize loss between the skeletal ROM of the residual limb to that of the prosthetic socket. The fenestrations offer a cooler socket environment as well as an avenue for tactile sensation on the residual limb while the prosthesis is worn. Though this subject had no active ROM of the residual limb, the socket fenestrations in the CRS design was chosen to take advantage of the features previously mentioned. The CRS socket design was modified to be loose enough to enable easy donning and doffing, and oversized anterior/posterior wings and a shoulder saddle were built into the socket to help support the weight of the prosthesis and house 2 EMG electrodes, over the pectoralis and trapezius muscles respectively. The initial socket was used to create a plaster mold and fabricate a flexible thermoplastic socket and rigid laminated frame.

During the fitting process, the subject was myo-tested to locate optimal EMG sites to use in controlling some of the functions of the prosthesis. As a result, two electrodes were incorporated into the anterior and posterior wings of the test socket so that the socket could be used during initial controls training in subsequent visits. A weighted “dummy arm” was affixed to the socket and a temporary harness was made. At the subsequent visit, the subject tried the test socket with the weighted dummy arm and wore it for approximately 7 continuous hours without any complaint of pain or discomfort. Minor adjustments were made to the alignment to satisfy the subject’s aesthetic concerns. At the subsequent visit the DEKA Arm was attached to the test socket and the controls set-up process was initiated.

Subject 1 had 10 device functions that were controlled by Inertial Measurement Unit (IMU) commands (1 IMU located on each foot), 2 functions controlled by EMG electrodes and two functions controlled by pneumatic bladder force sensitive resistors (Table 2). This subject chose to use the EMG signals to control the elbow flexion/extension function of the prosthesis. Though most other TH subjects in the Home study used EMG controls to operate the hand open/close function, this subject, due to prior experience using his existing prosthesis in dropping items in his prosthetic hand resulting from unintentional EMG signals, wanted to use a different control input for that function and selected IMU control for this purpose.

**Table 2:** Controls set up as of end of Part A.

	Subject 1 (H110)			Subject 2 (H225)		
DEKA Arm Level/Side	Right HC			Left SC		
Control Type	IMU		Other Control	IMU		Other Control
Foot	Left	Right		Left	Right	
**Prosthetic Action**						
Wrist Extension		Toe up*		Toe up		
Wrist Flexion		Heel up*		Heel up		
Wrist Supination		Roll out*		Roll out		
Wrist Pronation		Roll in*		Roll in		
Grip Toggle Forward	Roll out				Roll out	
Grip Toggle Backward	Roll in				Roll in	
Hand Open	Toe Up*				Heel up	
Hand Close	Heel Up*				Toe up	
Standby/OnAnd Mode Change (Hand Mode/Arm Mode)			Pneumatic bladder under sound arm			Pneumatic bladder under sound arm
Elbow Flexion			EMG	NA**		
Elbow Extension			EMG			
Elbow Internal Rotation	Roll out					
Elbow External Rotation	Roll in					
Endpoint Up	NA***				Toe up	
Endpoint Down					Heel up	
Endpoint Right					Roll in	
Endpoint Left					Roll out	
Endpoint Forward				Heel up		
Endpoint Back				Toe up		
Endpoint Elbow In				Roll in		
Endpoint Elbow Out				Roll out		
Activate/Deactivate Prosthesis	Power switch on prosthesis operated by sound hand			Power switch on prosthesis operated by sound hand		

* operated in both hand and arm modes

**Direct control of the elbow and humeral rotation is not available for SC configuration DEKA Arms

*** NA Endpoint control is not applicable for for humeral configuration DEKA Arms

**Fig 4:**
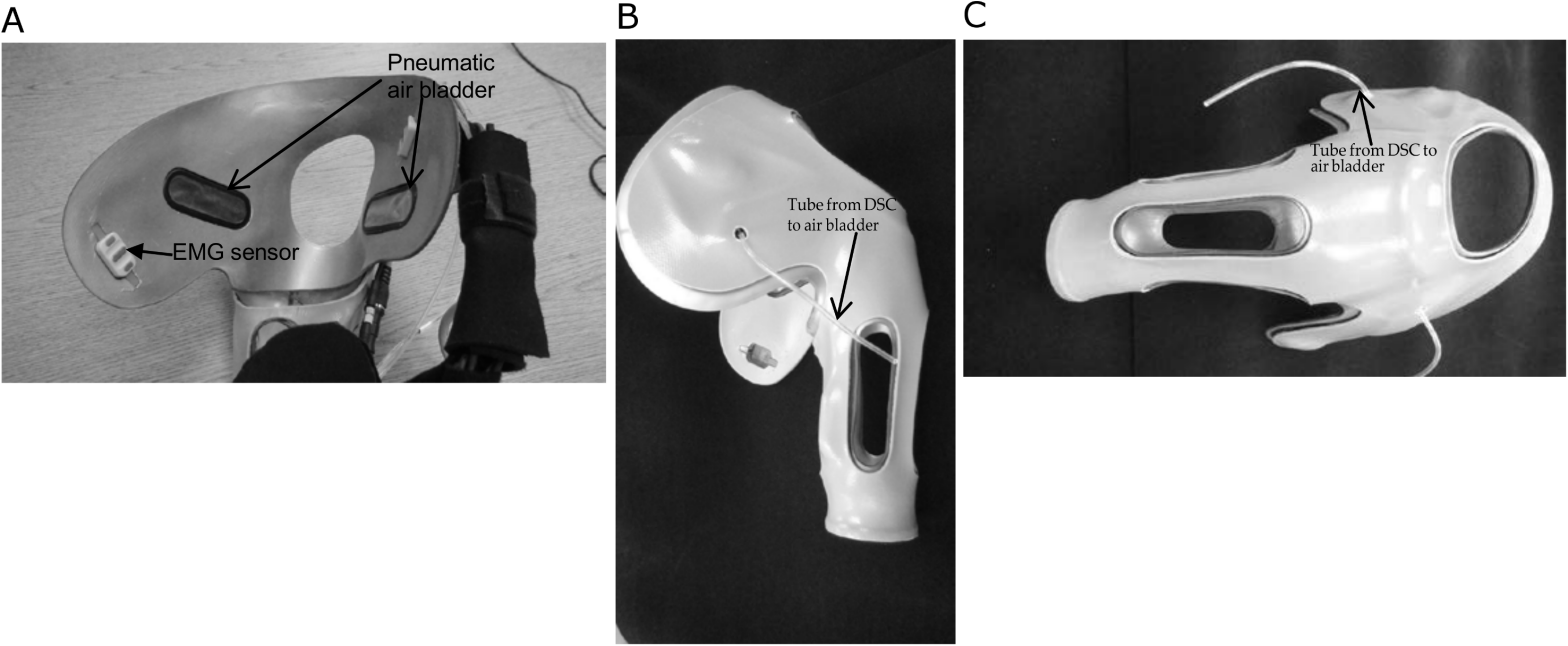
Subject 1. Final Prosthetic Socket Used for the Study: A. Interior View B. Posterior View, C. Lateral View.

**Fig 5:**
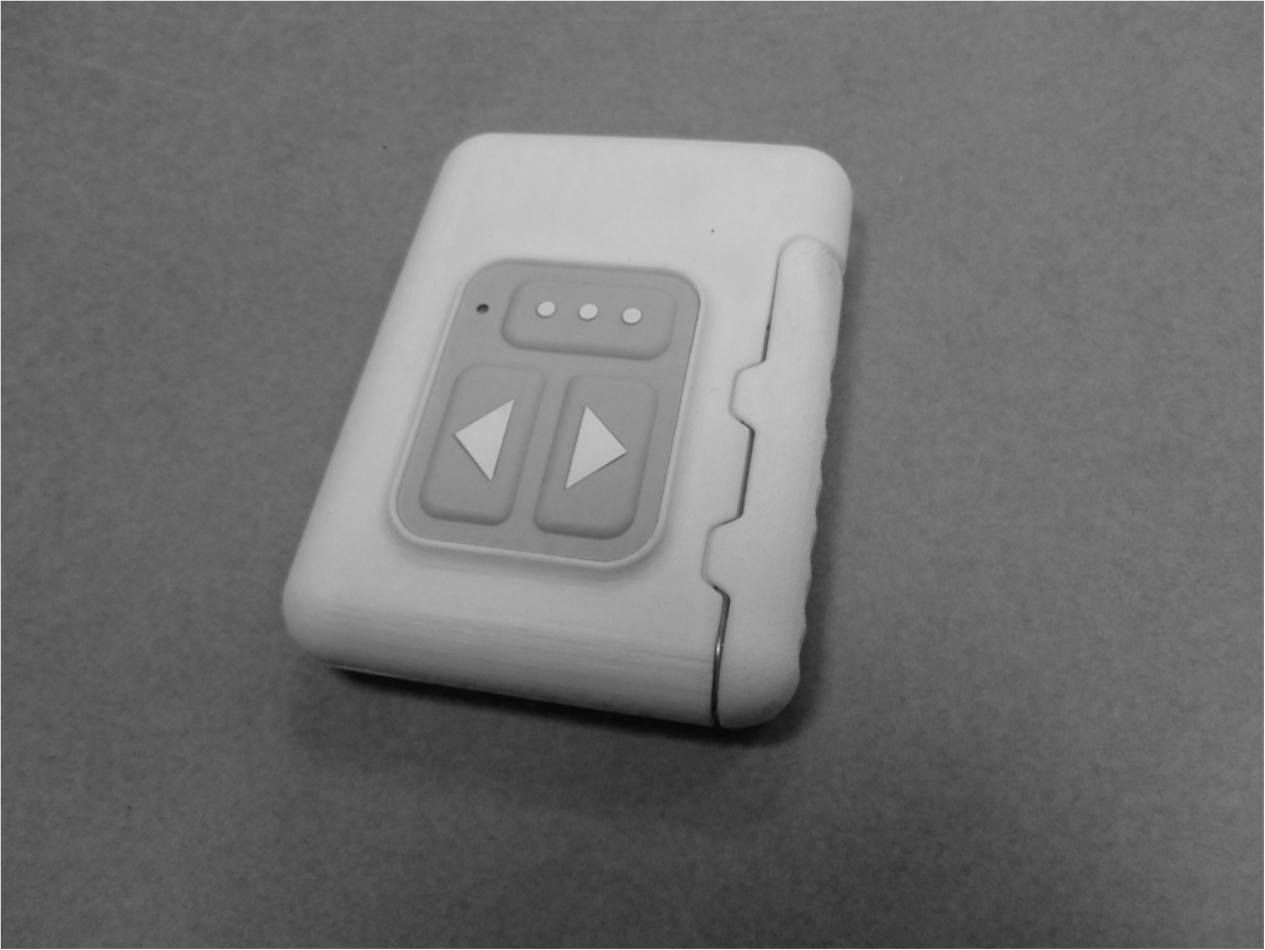
Dynamic Socket Controller (DSC).

**Fig 6:**
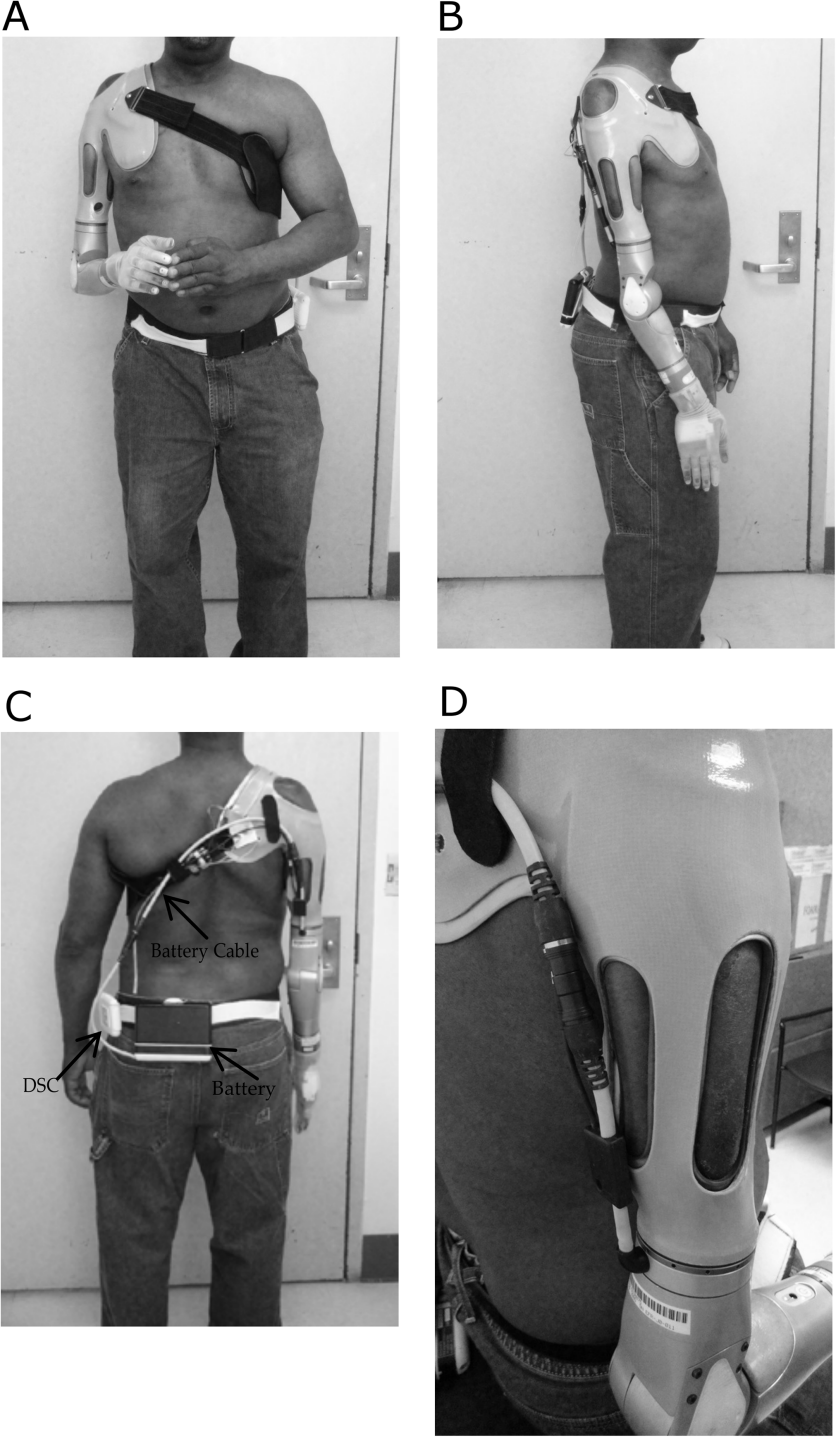
Subject 1 Wearing the DEKA Arm A. Anterior View B. Lateral View C. Posterior View D. Lateral view of socket showing fenestrations.

The subject trained in the laboratory with the test socket for several sessions. A definitive socket was fabricated and included a flexible thermoplastic inner socket with 2 embedded electrodes, as well as 2 embedded pneumatic bladders (placed on the chest and the scapula) which offered adjustability with the socket fit (Fig 4). A Dynamic Socket Controller (DSC) was introduced (Fig 5) and configured to allow the user to control the psi within the pneumatic air bladders embedded in the socket to modify comfort and pressures on the body within the socket Fig 6 shows the subject wearing the socket and DEKA Arm.

#### Study participation

Subject 1 had approximately 12 hours of prosthetic training which took place over 6 visits. His Part B participation took place over the course of approximately 6 months due to: time required to ship the arm to and from the research site; limitations in the subject’s availability to return to the study site for monthly visits due to his work schedule; and extra time required because the Arm was broken, being repaired or was in transit for repair. During Part B there were 6 instances where the Arm needed to be shipped for repair, 4 instances (related to replacement/repair of pneumatic bladders, arm lamination attachment, EMG wiring) were repaired on site. Two instances (a broken position sensor in the thumb, and a broken gear in the hand) required repair at DEKA.

#### Experience and outcomes

This subject was enthusiastic about the DEKA Arm and the fit of the prosthetic socket. At the end of Part A and Part B he indicated that he wished to receive a DEKA Arm in the future. At the end of Part A he rated his skill with the DEKA Arm as “good”, and rated the weight of the DEKA Arm as light and the socket as comfortable. He indicated that the weight of the Arm was light because the socket design allowed the weight of the prosthesis to be distributed over the entire shoulder girdle. He said that the comfort and fit of the socket were the best he had ever experienced saying that it had, “no slippage, no sliding. It’s in place all day long “.The subject described activities that he could do with the DEKA Arm that he could not do with his current prosthesis, but no activities that he could do with his current prosthesis that he could not do with the DEKA Arm. At the end of Part A he listed the following new activities he could do with the DEKA Arm: buttoning up a shirt; pulling up pants; zipping up a jacket; lifting an egg out of the carton without dropping it; and cooking. At the end of Part B he described some of the things he could only do with the DEKA Arm,

“In general, holding things, like I have a radio at work that I have to carry around. So holding that–I could actually pick it up off the desk and hold it and when I was doing something with my sound hand and carry it around. Sweeping, raking, cleaning dishes definitely—being able to hold dishes… holding them with the DEKA hand and then using, you know, scrubbing them with my sound hand.”

The subject stated that he preferred the control scheme on the DEKA Arm compared to his current prosthesis because the use of IMUs eliminated the need to use jerky upper body movements to operate the prostheses.

“It’s easier to control the DEKA Arm because, with my old prosthesis, I had to do like the old, jerky movements to get the Arm to unlock or do different things. With the DEKA Arm, I don’t have to do that. So it makes using it a lot easier because I don’t have to be in a certain position to jerk a certain way, or move my body a certain way to get it to move.”

At the end of Part B Subject 1 indicated that he liked the overall function of the DEKA arm, and the specific function of the hand, wrist, and elbow, more than he liked his own prosthesis. He also indicated that he liked the look and fit of the DEKA Arm and hand, as a whole, more than he liked his own prosthesis. This subject reported using the DEKA Dynamic Socket Controller throughout Part A of the study to change the pressure of air bladders within the socket, “after a number of hours of wearing it”, and that it was “like putting a little piece of cushion”, between his body and prosthesis. However, while at home in Part B he used it only “a few times”, such as one day when he was carrying packages.

This subject stated that he enjoyed using the DEKA Arm more than he enjoyed using his own device. When asked to comment on what he liked most about the DEKA hand he said,

“Having a hand that works. Having a hand that holds things. Having a hand that you could control as far as pressure on, you know, whatever you’re trying to grip or hold onto was the best part. A hand that opened wide enough to actually grip around something instead of having to pick it up and kind of try and shove it in there. That is definitely the best part.”

He indicated that the DEKA Arm was quite a bit necessary for maintaining his quality of life and independence and had contributed to improving his QOL and independence “quite a bit” since starting the study. At the End of Part B he commented,

“…it makes you more independent. I can do more things on my own for myself such as cooking, or cutting or, you know, (as compared to) having to struggle to do dishes, trying to do them one-handed. Or not having to work extra hard with my sound limb because I have two limbs like to carry groceries or tie things up or carry things out or carry things in, or move things around… ‘. I live alone, so I have to do a lot of things by myself, and having the DEKA Arm just makes it so much easier…. quicker to have that extra hand there to help out or to do things with.”

Table 3 shows the scores of the tests and measures at baseline, end of Part A and End of Part B. Standardized tests results for self-report measures confirmed Subject 1’s statements that he had less difficulty in performing activities when using the DEKA Arm as compared to his baseline scores as measured by the PSFS and UEFS. The UEFS use scale showed that he used the DEKA Arm to perform a greater proportion of activities at the end of both Parts A and B. Subject 1’s CRIS-CAT extent of participation scores stayed fairly constant throughout the study. However, his perceived limitations and satisfaction with participation score were greater at the end of Part A as compared to baseline and the end of Part B. Whereas, QOL scores were slightly higher at the end of Part B. Subject 1 rated his satisfaction with the DEKA Arm higher than the satisfaction with his own prosthesis. The subject’s reports of pain were low and largely consistent at all assessment points. Subject 1’s scores on performance measures were better with the DEKA Arm as compared to his own prosthesis. Dexterity, as measured by the JTHF improved from baseline to the end of Part B for 6/7, scores of the UNB prosthetic skill and spontaneity were unchanged, and scores of the AM-ULA improved from baseline to End of A and End of B.

**Table 3:** Comparison of outcomes at baseline, end of laboratory training (A), end of home use (B).

	Subject 1			Subject 2		
**Self-report measures**	**Baseline**	**End of A**	**End of B**	**Baseline**	**End of A**	**End of B**
PSFS	2.3	3.7	5.0	1.8	5.2	NT*
UEFS	49.5	44.3	45.3	57.6	41.4	NT
UEF use	0.4	0.8	0.8	0.0	0.5	NT
QuickDASH	20.5	20.5	22.7	63.6	72.7	NT
CRIS-CAT						
Extent	50.0	53.0	50.0	49.0	27.0	NT
Perceived Limitations	51.0	59.0	52.0	42.0	38.0	NT
Satisfaction	47.0	53.0	48.0	41.0	40.0	NT
Quality of Life Scale	5.5	5.4	5.8	4.6	3.9	NT
TAPES Satisfaction	2.8	4.3	4.3	NT	3.3	NT
Wong-Baker Pain Scale	1.0	2.0	1.0	5.0	4.0	NT
**Performance measures**						
JTHF: Writing	0.09	0.13	0.30	NT	0.50	NT
JTHF: Page Turning	0.01	0.03	0.05	NT	0.00	NT
JTHF: Small items	0.01	0.03	0.06	NT	0.00	NT
JTHF: Feeding / Eating	0.13	0.00	0.01	NT	0.00	NT
JTHF: Light Cans	0.05	0.02	0.13	NT	0.02	NT
JTHF: Heavy Cans	0.09	0.00	0.13	NT	0.02	NT
UNB: Skill	2.4	2.5	2.5	NT	2.1	NT
UNB: Spontaneity	2.5	2.5	2.5	NT	2.3	NT
AM-ULA	8	16	15	*	11	NT

*NT = not tested

### Subject 2

#### Prosthetic fitting and controls set-up

Although the amputation was at the transhumeral level, the research clinicians, in consultation with the subject, decided against fitting with the TH configuration. Several factors contributed to this decision. Among these factors was the presentation of the non- surgically stabilized subluxed shoulder, the level of pain in the shoulder, the anticipated distractive forces the shoulder would be subject to if a typical TH socket configuration and subsequent axial load were applied to the residual limb. Therefore, the subject was fit with an SC DEKA Arm. The challenge faced with fitting a SC socket configuration over a TH residual limb is the location of the shoulder joint in relation to the torso and the resultant excessive asymmetry with the body, functional alignment issues, and the fit of the subjects clothing. The prosthetic team thought that these challenges might be offset by the 3 degrees of shoulder motion to be regained by using the SC configuration; shoulder flexion and extension, shoulder abduction and adduction and transhumeral internal and external rotation.

**Fig 7:**
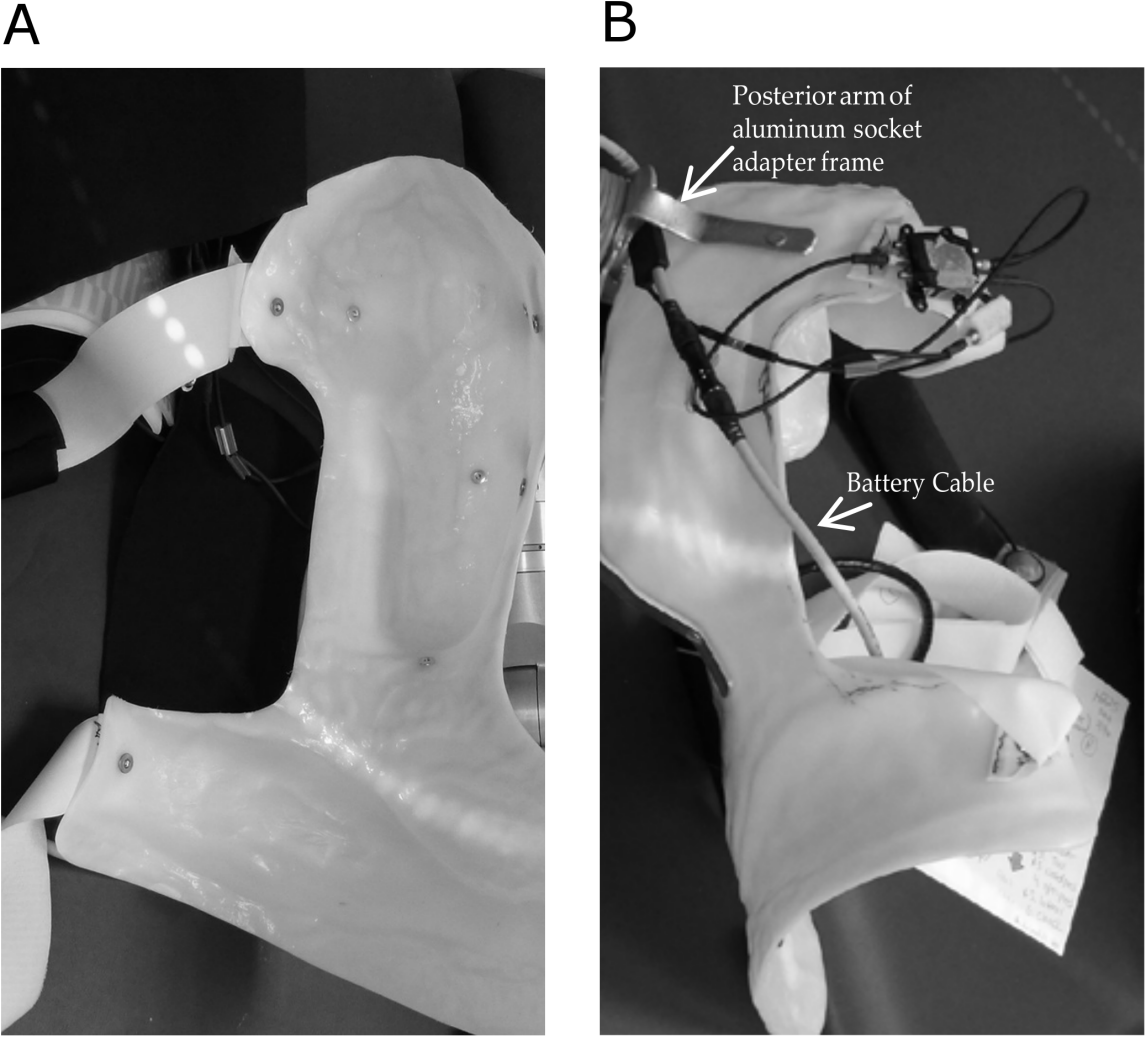
Subject 2 left transhumeral amputee fit with an SC DEKA arm. A. Interior View. B. Posterior View.

Fitting and set-up of prosthetic controls for this subject took place over the course of 3 visits. A modified X-frame socket design was used, consisting of a flexible thermoplastic inner socket and a rigid plastic outer frame (Fig 7). The trim lines of the inner socket encompassed the upper trapezius, glenohumeral joint and transhumeral residual limb, and extended along the ipsilateral torso to just distal of the iliac crest to carry the weight of the DEKA arm SD prosthesis. The proflex plastic acted as a flexible interface between the rigid frame and the subjects’ body, extending slightly beyond the trimlines of the rigid frame, and also wrapped over the top of the shoulder and upper trapezius, where the rigid frame did not extend, creating a flexible saddle to aid in suspension of the prosthesis. The rigid frame of the socket wrapped posteriorly around the scapula and around the proximal border of the spine of scapula (Fig 7). The anterior proximal arm of the X-frame rested on the ipsilateral pectoralis musculature, just distal to the clavicle. The distal wings of the x-frame wrapped around the waist from ipsilateral anterior superior iliac spine to the posterior superior iliac spine and rested on the iliac crest. Straps held the X- frame onto the torso of the subject by utilizing a pad along the torso on the contralateral ribcage, waist and pelvis. The socket adaptor consisted of a 4 armed aluminum bracket that both reinforced the frame and dispersed the weight of the arm through the X-frame. This design allowed the weight of the prosthesis to be distributed over the ipsilateral shoulder, ipsilateral pelvis and the contralateral waist area. A neoprene liner was added to the inside of the X-frame over the spine of the scapula, and clavicle for added comfort.

**Fig 8:**
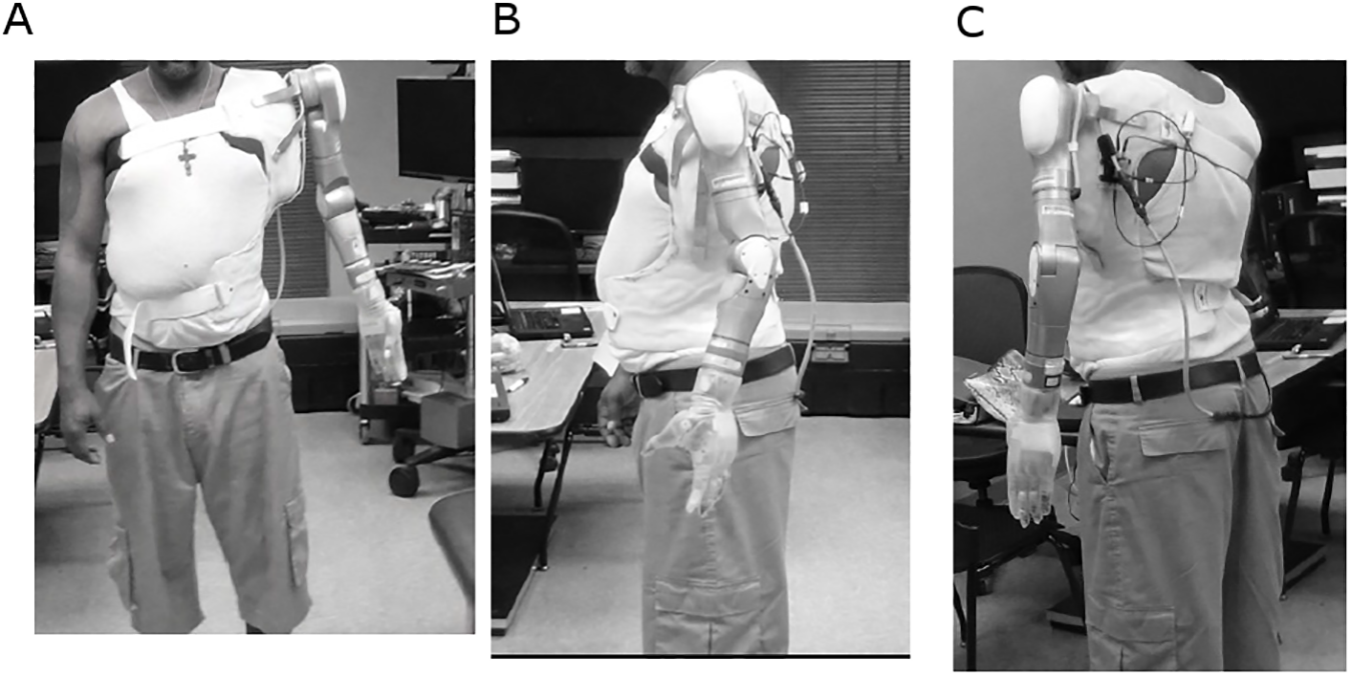
Subject 2 wearing the DEKA Arm and socket. A. Anterior View, B Lateral View, C. Posterior View.

Subject 2 did not elicit sufficient myoelectric signals to incorporate the use of EMG control. Therefore, this participant had 16 device functions that were controlled by IMU commands (1 IMU located on each foot), and two functions (mode select/standby controlled by pneumatic bladder force sensitive resistors (Table 2). The use of IMU control made it possible to operate the arm without the gross body movements needed to create cable tension or the need to locate, train and develop control over multiple myosites. Fig 8 shows the subject wearing the prosthetic socket and DEKA Arm.

#### Study participation

Subject 2, who used an SC configuration, had approximately 30 hours of training over the course of 15 visits which included 5 community outings. This subject withdrew from Part B after 3 weeks of home use. He explained his reasons for withdrawal: family problems, including a death in his family; personal problems related to his TBI; triggering of his PTSD symptoms, saying he “just didn’t want to use the Arm”; and that he was concerned about the responsibility of keeping the Arm safely in his home.

#### Subject experience and outcomes

At the end of Part A, Subject 2 indicated that he wished to receive a DEKA Arm in the future, saying that it, “opens up the world again to me.” His enthusiasm about the device was evident when he said, “I have my arm back. I’m whole.” He rated his skill with the DEKA Arm as “excellent”, despite commenting that some of the movement trajectories in Endpoint control “didn’t feel normal”.

At the end of Part A Subject 2 indicated that he was able to do things with the DEKA Arm that he could not do without a prosthesis, including: opening a bottle, holding a can, opening a can, pouring a drink, holding a pot. However, he acknowledged that using the DEKA Arm for certain activities, such as tying a shoe or putting on a sock, made the activities more difficult, explaining, I’m just accustomed to doing it with one (hand), I can do it quick and fast.” As a non-prosthesis user, he acknowledged that he had to “retrain” himself to use two hands. At the end of Part A, he indicated that the device was “a little heavy” and he rated the socket comfort as “tolerable”.

The subject’s overall impression of the DEKA Arm after 3 weeks in Part B was that it was “awesome” and he answered “of course” when asked if he would want a DEKA Arm if it were available in the future, saying he would like to be the “first in line to get one”. He also indicated that the weight of the DEKA Arm and the cables and straps, which went across his back, were the things that he liked least about it. The weight of the Arm, he said, was a factor in how long he could wear the Arm, but only when he was “having severe phantom pain”. He estimated that he could wear the Arm “for a lengthy amount of time,” approximately “eight hours”, if he “had to “. He indicated that he thought that the straps would be more comfortable if they were wider, saying, then “they wouldn’t move as much and they would be easy to grab and put together. It would feel more secure on the body and I’d feel more secure with it.” However, the subject did not return to the study site for his 4 week reassessment, at which time, the prosthetists might have been able to make modifications to the straps.

When the subject withdrew from Part B, he rated the DEKA Arm as moderately necessary for maintaining his quality of life, and slightly necessary for maintaining his independence. He indicated that the DEKA Arm had contributed moderately to improving his QOL, and independence since beginning the study. He explained that he was able to do more things including: opening up a cabinet, holding two hands on a garbage bag, holding the garbage bag with that arm, opening up the door. He said,

“I definitely could perform activities and get things done with no help from anybody. A perfect example of that was zipping up my jacket, which is something that I normally need help with, but the DEKA Arm allowed me to be independent with that. I am normally not too dependent though.”

The subject indicated that he did not wear the DEKA Arm at home as much as he expected, in part, because of self-consciousness and hypervigilance. He explained,

“I would say, (I was) nervous, I was cautious about wearing it…around other people….

…. I, honestly, I didn’t wear it around other people that much, for the fact of the matter of the expense of the Arm and you knowing it and, you know, I just, my PTSD just always has me on guard when I’m around people, or people may be looking at me or something like that. And I apologize–that’s in my head, that’s just me.”

…, I’m constantly on guard. And having it here (the DEKA Arm), you know, I didn’t have it out–I was always putting it away every night, locking it up… …and making sure and going through all of that every day and making sure my house is locked up…”

The subject did comment, when asked what surprised him about using the DEKA Arm at home, that it was difficult for him to acclimate to the use of the IMU controls, saying:

“…when I was standing and certain things I needed to do, you know, I had to reset my feet and to be prepared, it was like, go to the sink, stop. Do what I had to do, you know? Stop. Instead of just doing it fluently when I was at the sessions.”

He also reported some inadvertent movements as a result of not paying attention to his foot movements:

“. you just get up and you just, you’re not thinking, and you just get up and move. Well, once you, if you’re doing something, say if I was picking up a Gatorade bottle and I just wanted to stand up and move, the controls are still live, the IMUs are still alive, so it tends to do what I what I don’t want it to do.”…., I’d have to have the bottle, stop, stand up, stop, you know, how I say it, “freeze it,” put it in neutral and then move.

Results of self-report measures administered at the end of Part A show less perceived difficulty (as compared to baseline) in activity performance, as measured by the PSFS and UEFS. He reported engaging the prosthesis in about 50% of activities of the UEFS. However, the subject’s self- reported disability, as well as scores of all 3 CRIS-CAT scales and QOL decreased between baseline and the end of Part A. Although his performance scores cannot be compared to his own baseline performance (because he was not a prosthesis user), Subject 2 scored lower on dexterity tests, prosthetic skill, spontaneity, but slightly higher on activity performance as compared to Subject 1. Subject 2 was not tested at the end of Part B because of his early termination. Although he did not complete a Wong Baker scale at the time of his termination from the study, the subject indicated in his exit interview that his phantom limb pain was a level 10, and he associated this with his stress level saying:,

“… it’s kind of hard to describe, because it’s neurological and if I get into a calm state, I don’t have that much pain. But up, moving around, and if something stresses me, I tend to get it more… And when I say that its level 10, that means my meds are working but it’s actually stronger… My 10, I don’t think anybody, even with two arms, would be able to deal with that pain level.”

## Discussion

This paper described two very different examples of prosthetic fitting for transhumeral amputees with brachial plexus injuries. The first example was for an amputee with a shoulder arthrodesis who was treated with an HC DEKA Arm, while the second example was for an amputee with a subluxed shoulder who was treated with a SC DEKA Arm. The prosthetic prescription was determined in large part based upon the status of the amputee’s shoulder joint (whether it had been stabilized surgically or not), potential to tolerate the weight of prosthetic suspension on the residual limb, and the pain level in the shoulder joint.

Both subjects found the prosthetic socket comfortable to wear, reported functional benefits from use, and expressed a desire to receive a DEKA Arm in the future. However, Subject 1’s experience was clearly more successful. Subject 1 was more satisfied with the HC DEKA Arm, enjoyed using it more, preferred the control scheme, and had better function with the DEKA Arm as compared to his own prosthesis. His enthusiasm did not seem to be tempered by the fact that he returned the DEKA Arm to the site 6 times for repair. He also reported being less limited and more satisfied with his role functioning at the end of Part A and reported better reported QOL at the end of Part B. Although this Subject was fit with a DSC and was enthusiastic about its use during the in-laboratory training, he rarely used it during his home experience, suggesting that the DSC was a minor, or insignificant factor in this case. Subject 1 was an experienced myoelectric prosthesis user and he maintained the same EMG control patterns in the DEKA Arm for elbow flexion/extension as he had used in his personal prosthesis. This control familiarity may have been a factor in his acceptance of the DEKA Arm.

Subject 2 however, withdrew from the study early without completing all home use activities, citing personal problems with an exacerbation of his PTSD symptoms and phantom limb pain and a negative desire to use the DEKA Arm due, in part, to self-consciousness and hypervigilance. Although Subject 2 was enthusiastic about the SC Arm at the end of Part A and was reported less functional disability as compared to baseline, he found the experience and responsibility of taking the device home more challenging, and was surprised at the difficulty of acclimating to the use of IMUs at home. While this Subject provides an illustrative example of how a TH amputee with brachial plexus injury could be fit with an SC DEKA Arm, this particular subject did not appear to be a good candidate for the device, in large part because of unresolved mental and physical health issues which interfered with his device usage at home. This phenomenon is concordant with findings reported in a case study of another study subject who was deemed an inappropriate candidate for the SC DEKA Arm. [30] Although Subject 2 did not manifest problems with safety awareness or judgment that were evident in the previously reported case, it was clear that his PTSD symptomatology impacted his ability to use the DEKA Arm in a community setting. Clearly, the timing of study participation and issues in his personal life (death in the family etc.) were a factor in his decision to withdraw from the study. It is possible, that this subject might be a candidate for the SC DEKA Arm, if personal circumstances were different and his health concerns were ameliorated.

One of the disadvantages of using an SC DEKA Arm on anyone other than those with scapulothoracic amputation is that the shoulder joint of the device must be placed lateral to anatomical shoulder (the prosthetic shoulder is sometimes placed inferior to the anatomical shoulder, if the residual limb is short enough to allow it, in order to minimize body width asymmetry and resulting functional issues). This creates an ‘expensive cosmetic issue,’ particularly for those with residual limbs that widen the placement even further, in exchange for active shoulder function. This creates substantial body asymmetry and may have contributed to Subject 2’s sense of self-consciousness about wearing the device in public. The decision to treat this Subject with an SC, rather than an HC DEKA Arm was made to allow greater range of motion and functional use, and minimize stresses on his unstable shoulder joint. However, we cannot say whether or not this subject had he had a shoulder disarticulation, rather than a TH amputation, would have had a better cosmetic outcome and how this may have impacted his willingness to use the device in public. We also cannot say whether this Subject would have had a better outcome, or been able to tolerate wearing an SC if treated similarly to Subject 1. Subject 2 was not using a prosthesis at baseline. While abandonment of prostheses is common amongst persons with similar injuries, use of a personal prosthesis may also be a strong predictor of ultimate acceptance of a DEKA Arm. [31] Indeed, our analyses of home study data suggest that prior prosthesis use is a strong predictor of study completion.

There are several important limitations to the interpretation of results of our study- as they pertain to the “success” of the DEKA Arm and appropriateness of prescription of the device for these subjects. This was a case series that reported on two subjects from a convenience sample who were willing and able to participate in the study. Although we believe that many findings and lessons learned from these case examples are instructive for other patients with transhumeral level amputation and brachial plexus injury, we recognize that these two examples cannot be generalized to all similar patients. The need for frequent repair did not temper Subject 1’s enthusiasm for the device, perhaps because he felt that the trade-off between improved function and inconvenience was worthwhile. However we cannot say whether other persons with similar case histories and limitations would share this view.

Another limitation is that our study had an accelerated training program with no additional training after discharge to home use. This training program may not have adequately addressed the needs of Subject 2, who might have benefited from additional time to acclimate to using the DEKA Arm in public with the support of study staff. Another limitation is that subjects were not able to keep the DEKA Arm at the end because it was not yet commercially available. Thus they were training to use and acclimate to a device that they had no guarantee of ever obtaining. It is possible that Subject 2 would have been more motivated to continue study activities if he knew that he would be able to keep the device at the end.

## Conclusions

This case series described prosthetic fitting, control set-up, and outcomes of two persons with transhumeral amputation and brachial plexus injuries who participated in the VA Home Study of the DEKA Arm. Two different prosthetic approaches offering multiple powered degrees of freedom are illustrated for patients with moderate length residua, the first an HC DEKA Arm for a patient with a shoulder arthrodesis and the second an SC DEKA Arm for a patient with a subluxed shoulder. The DEKA Arm allows more powered degrees of freedom than other devices currently commercially available, and utilize IMU foot controls to expand the control options available to prosthesis users. Given the challenges in prosthetic fitting and acceptance in this patient group the results suggest that the DEKA Arm, a newly FDA approved prosthesis, could be considered as options for patients with TH amputation and brachial plexus injury. We found that the HC device was well accepted, however results with the SC DEKA Arm were mixed. Further research is needed to explore the acceptability of the SC DEKA Arm for persons with brachial plexus injury and subluxed shoulders. Clinicians should be aware that clinical considerations, such as impaired cognitive status and prior history of prosthesis abandonment may be factors that could interfere with device acceptance. The case details on fitting and control set-up and the influence of cognitive and psychological functioning on outcomes may be instructive to clinicians considering prosthetic options for future patients.

## Supporting information

S1 File Supplemental survey questions. Supplemental survey questions.docx
(DOCX)
