# Supplementary material for: Use of the DEKA Arm for amputees with brachial plexus injury: A case series
Source: PLoS One. 2017 Jun 19;12(6):e0178642. doi: 10.1371/journal.pone.0178642 (PMC5476237; doi:10.1371/journal.pone.0178642)
Supplement: S1 File — Supplemental survey questions.docx (DOCX) [file pone.0178642.s001.docx]

**Part A Participant Survey End of Study**

- Please check the box that best describes your overall skill in using the DEKA Arm as of

today ? (“Very Poor”, “Poor”, “Fair”, Good”, or “Excellent”)

- Would you want to receive a DEKA Arm in the future? (“No”, “Yes”, or “Maybe”):

# Please explain why or why not:

- Do you feel that the amount of training you had with the DEKA Arm was not enough, too

much, or just right?

# Please comment on the comfort and fit of the socket:

# As of today, please rate the comfort of the socket interface with the DEKA Arm

# after wearing it for one hour?

# (“Could not tolerate, needed to remove”, “Uncomfortable”, “Aware of presence,

# tolerable”, “Comfortable”, or “Very comfortable, could wear indefinitely”):

- Have you experienced any changes in “wearability” of the Arm and socket since you

began the study? (For example, does it feel lighter, heavier, more comfortable, less

comfortable, etc.?)

- As of today, please rate your perception of the weight of the DEKA Arm System after

wearing it for one hour?

(“Very light”, “Light”, “A little light”, “Heavy”, or “Very heavy”):

- Are there activities that you would prefer doing with the DEKA Arm rather than your

current prosthetic arm?

Please describe:

- Are there activities that you would prefer doing with your current prosthetic arm rather than the DEKA Arm?

Please describe:

**Part B Participant End of Study Survey**

- Please check the box that best describes your overall skill in using the DEKA Arm as of

today? (“Very Poor”, “Poor”, “Fair”, “Good”; or “Excellent”):

- Would you want to receive a DEKA Arm in the future? (“No”, “Yes”, or “Maybe”):

Please explain why or why not:

- Please describe any changes in “wearability” of the Arm and socket since you began the

study? (For example, does it feel lighter, heavier, more comfortable, less comfortable, etc.?)

- As of today, please rate your perception of the weight of the DEKA Arm system after wearing it for one hour?

(“Very light”, “Light”, “A little light”, “Heavy”, or “Very heavy”):

- As of today, please rate the comfort of the socket interface with the DEKA Arm after wearing it for one hour?

(“Could not tolerate, needed to remove”, “Uncomfortable”, “Aware of presence,

tolerable”, “Comfortable”, “Very comfortable, could wear indefinitely”):

- Please compare the DEKA Arm to your primary prosthesis, if you have one.
  - I like the function of the DEKA hand better.
  - I like the controls of the DEKA Arm system better.
  - I like the weight of the DEKA Arm system better.
  - I like the function of the DEKA wrist better.
  - I like the look of the DEKA hand better.
  - I like the look of the whole DEKA Arm system better.
  - I like the socket fit and general comfort of the DEKA Arm more.
  - I enjoyed using the DEKA Arm more.
  - For DEKA elbow users: I like the function of the elbow better.
  - For DEKA shoulder users: I like the function of the shoulder and whole arm better.
  - I like the overall function of the DEKA Arm system better.
- Are there activities that you would prefer doing with the DEKA Arm rather than your

current prosthetic arm?

Please describe:

- Are there activities that you would prefer doing with your current prosthetic arm rather than the DEKA Arm?

Please describe:

- How necessary is the DEKA Arm to you for maintaining your quality of life?

(“Not at all”, “Slightly”, “Moderately”, “Quite a bit”, or “Extremely”):

- How necessary is the DEKA Arm to you for maintaining your independence?

(“Not at all”, “Slightly”, “Moderately”, “Quite a bit”, or “Extremely”):

- Since starting the home study, please rate how much using the DEKA Arm has contributed to improving your quality of life?

(“Not at all”, “Slightly”, “Moderately”, “Quite a bit”, or “Extremely”):

Please explain your answer.

- Since starting the home study, please rate how much using the DEKA Arm has contributed to improving your independence?

(“Not at all”, “Slightly”, “Moderately”, “Quite a bit”, or “Extremely”):

Please explain your answer.
